# Neuroprotective and therapeutic effects of calcitriol in rotenone-induced Parkinson’s disease rat model

**DOI:** 10.3389/fncel.2022.967813

**Published:** 2022-09-16

**Authors:** Alshimaa Magdy, Eman A. E. Farrag, Shereen Mohamed Hamed, Zienab Abdallah, Eman Mohamad El Nashar, Mansour Abdullah Alghamdi, Amira A. H. Ali, Marwa Abd El-kader

**Affiliations:** ^1^Department of Medical Biochemistry, Faculty of Medicine, Mansoura University, Mansoura, Egypt; ^2^Department of Pharmacology, Faculty of Medicine, Mansoura University, Mansoura, Egypt; ^3^Department of Medical Histology, Faculty of Medicine, Mansoura University, Mansoura, Egypt; ^4^Department of Medical Physiology, Faculty of Medicine, Mansoura University, Mansoura, Egypt; ^5^Department of Anatomy, College of Medicine, King Khalid University, Abha, Saudi Arabia; ^6^Department of Histology and Cell Biology, Faculty of Medicine, Benha University, Benha, Egypt; ^7^Genomics and Personalized Medicine Unit, College of Medicine, King Khalid University, Abha, Saudi Arabia; ^8^Department of Human Anatomy and Embryology, Faculty of Medicine, Mansoura University, Mansoura, Egypt; ^9^Institute of Anatomy ll, Medical Faculty, Heinrich Heine University, Düsseldorf, Germany

**Keywords:** rotenone, Parkinson’s, Sirt1, NF-_k_B, autophagy, vitamin D

## Abstract

Parkinson’s disease (PD) is the second most common neurodegenerative disease. Treatment of PD is challenging, as current treatment strategies are only symptomatic and do not stop disease development. Recent studies reported neuroprotective effects of calcitriol in PD through its antioxidant and anti-inflammatory properties. The exact pathomechanisms of PD are not yet fully understood. So, investigation of different molecular pathways is challenging. Sirtuin-1 (Sirt1) modulates multiple physiological processes, including programmed cell death, DNA repair, and inflammation. Furthermore, defective autophagy is considered a key pathomechanism in PD as it eliminates protein aggregation and dysfunctional cell organelles. The present study investigated the involvement of autophagy and Sirt1/NF-κB molecular pathway in rotenone-induced PD and explored the protective and restorative effects of calcitriol through these mechanisms. Therefore, behavioral tests were used to test the effect of calcitriol on motor disability and equilibrium. Furthermore, the histological and neuronal architecture was assessed. The expression of genes encoding neuroinflammation and autophagy markers was determined by qPCR while their protein levels were determined by Western blot analysis and immune-histochemical staining. Our results indicate that behavioral impairments and dopaminergic neuron depletion in the rotenone-induced PD model were improved by calcitriol administration. Furthermore, calcitriol attenuated rotenone-induced neuroinflammation and autophagy dysfunction in PD rats through up-regulation of Sirt1 and LC3 and down-regulation of P62 and NF-κB expression levels. Thus, calcitriol could induce a neuro-protective and restorative effect in the rotenone-induced PD model by modulating autophagy and Sirt1/NF-κB pathway.

## Introduction

Parkinson’s disease (PD) is a progressive age-related neurodegenerative disorder that affects 12% of the population over 65 years. Its incidence is expected to double by 2030 due to the global increase in the age and lifespan (Hegarty et al., 2020).

The exact mechanism of PD has not yet been fully explained. However, PD is considered a multifactorial disease that includes a variety of genetic and environmental factors by which oxidative stress, neuroinflammation, and mitochondrial dysfunction play an essential role in the pathogenesis of PD (Hattingen et al., 2009). The available therapeutic approaches for PD are mainly symptomatic and can only improve the clinical symptoms; however, they cannot stop the PD progression (Zhao et al., 2019).

Recently, various studies have shown the protective role of Sirtuin-1 (Sirt1), one of the seven Sirtuins, in neuroinflammation. Sirt1 exerts an important effect in modulating multiple physiological processes, including programmed cell death, DNA repair, inflammation, and oxidative stress (Jiao and Gong, 2020). Several observations suggest that Sirt1 could induce a beneficial effect in PD models. Numerous *in vivo* and *in vitro* studies have found that upregulation of Sirt1 provided a protective role in various experimental models of PD (Zhang et al., 2018).

In addition, PD is correlated with protein aggregation in dopaminergic neurons (DA) (Prasad and Hung, 2020). As autophagy eliminates protein aggregation and accumulation of dysfunctional cell organelles, a defective autophagy-lysosome system is considered a key pathomechanism in PD (Monaco and Fraldi, 2020). Therefore, it is suggested that autophagy activation is one of the important therapeutic strategies in PD (Lin et al., 2019).

Thus, Sirtuins and functional autophagy appear to play a protective role against environmental stresses, including nutrient stress (Kroemer et al., 2010; Kim et al., 2013). Under cellular stress, Sirt1 activity is stimulated, autophagy-related proteins are deacetylated, and consequently, autophagy is enhanced. The characteristic feature of autophagy in mammals is increased microtubule-associated protein 1A/1B light chain 3 (LC3) expression. Importantly, increased Sirt1 expression is convenient for induced autophagy by deacetylation of LC3 (Lee et al., 2008).

Nuclear factor-κB (NF-κB) transcription factor is a central regulator of inflammation and apoptosis. NF-κB is involved in the aging and pathogenesis of a wide variety of neurodegenerative disorders. Recent studies highlight the role of *NF-*κ*B* subunits in PD and indicate that dysregulation of this transcription factor may be associated with the onset of PD (Bellucci et al., 2020).

The relationship between calcitriol, the active form of vitamin D (VD), and PD has attracted consideration (Newmark and Newmark, 2007). Muscular and motor impairments, which could induce detrimental effects on motor behavior, were reported in VDR-knockout mice (Burne et al., 2005). Interestingly, Wang et al. (2016) found that decreased serum calcitriol levels could increase the risk of PD. Furthermore, patients with PD with lower calcitriol levels may exhibit more severe symptoms compared to patients with normal calcitriol levels (Sleeman et al., 2017). Recently, limited studies have discussed the neuroprotective effects of VD in experimentally-induced PD models. VD was reported to reverse behavioral changes and improve the decreased DA content in the striatum of 6-OHDA induced PD model. Furthermore, VD significantly improved the expression of tyrosine hydroxylase (TH), dopamine transporter (DAT), as well as brain-derived neurotrophic factor (BDNF) and reduced the expression of mitochondrial injury, oxidative stress (nitrite contents, H_2_O_2_, and lipid peroxidation) and inflammatory markers (TNF-alpha, IL-Iβ) markers (Lima et al., 2018; Araújo de Lima et al., 2022; Bayo-Olugbami et al., 2022). However, none of the previous studies discussed the effect of VD on Sirt1 and autophagy in the experimentally-induced PD models.

The present study was designed to investigate for the first time, the association of Sirt1/NF-κB molecular pathway and autophagy in the midbrain of the rotenone-induced PD model. The study also aimed to explore and compare the protective and restorative effects of calcitriol on the induced PD model.

## Materials and methods

### Drugs

Rotenone (ROT) (Sigma-Aldrich: St. Louis, MO, United States) was dissolved in dimethylsulfoxide (DMSO) at 50X stock solution and then diluted in sunflower oil to obtain a final concentration of 2.5 mg/ml. To induce PD, ROT (2.5 mg/kg/day) was injected intraperitoneally (ip) for 4 weeks as described by Ojha et al. (2016). Treatment with ROT was shown to induce Lewy body-like inclusions, electrophysiological alteration in striatal neurons, and irreversible dopaminergic neuronal loss in the midbrain in addition to behavioral deficits (Vehovszky et al., 2007; Costa et al., 2008; Zagoura et al., 2017). Calcitriol (Sigma Aldrich: St. Louis, MO, United States) was diluted in propylene glycol and administered (1 μg/kg/day, i.p) according to the study by Cass et al. (2014).

### Experimental animals

Thirty adult male Sprague Dawley rats (age 10-12 weeks; weight, 260 ± 20 gram) were purchased from the Medical Experimental Research Center (MERC), Faculty of Medicine, Mansoura University, Egypt. Rats were housed under temperature-controlled conditions and a 12 h/12 h light-dark cycle. All experiments were approved by the ethical committee, Faculty of Medicine of Mansoura University, and followed the guidelines for the care and use of laboratory animals of the National Institutes of Health. All efforts were made to decrease the animal number and suffer.

After one week of acclimatization, rats were randomly divided into five groups of six rats each: a control group injected with vehicle (DMSO + sunflower oil) once a day for 4 weeks, another group of rats was injected with ROT as described above for 4 weeks to induce PD (Ojha et al., 2016), a third group was injected with calcitriol once daily for 4 weeks. The fourth group was injected with calcitriol 30 minutes prior to ROT injections (calcitriol sim ROT) to test the prophylactic effect of calcitriol and, finally, a fifth group was injected with ROT for 4 weeks followed by calcitriol administration for 8 days (calcitriol after ROT) to test the therapeutic potential.

The rats were trained for two consecutive days. One day before sacrifice, the experiments were conducted.

### Open-field test

To evaluate motor performance, exploratory behavior, and anxiety in rats, the open field was used. The open field apparatus is made of white plywood with dimensions: 72 × 72 cm floor and 36 cm high walls. One of the walls and floor were constructed of clear Plexiglas, so rats could be tracked inside the apparatus. The floor was divided by blue lines into sixteen squares (18 × 18 cm), with a central square in the middle of the open field. On test day, rats were moved to test rooms within their home cages and were adapted to the testing room prior to testing. Rats were quietly placed by an experienced investigator in one of the four corners of the apparatus and were allowed for 5 minutes to explore the open field, then the rats were returned to their home cages. Between every two tests, the open field was cleaned with 70% ethanol and left to dry. The behavior in the open field was documented by a digital camera (Adel et al., 2021). Different parameters such as distance traveled, immobile latency, and rear were used to assess motor performance, bradykinesia, and exploratory behavior, respectively (Askar et al., 2019). Measurements anxiety; the number of central square participants, grooming, rearing and stretch attending postures were used (Smolinsky et al., 2009; Kamel and El-lethey, 2011).

### Rotarod test

Animals were monitored for motor coordination and balance using a rotarod apparatus composed of a cylindrical arrangement of 3 cm diameter thin steel rods which is rotating constantly at a speed of 20 rpm). First, the animals received two sessions of training: 5 min each separated with a 10 min gap between the two sessions, to allow the rats to habituate to maintain their posture on the rotarod. After training, the animals were tested by allowing them to move over the rotarod. The fall time was recorded with an endpoint limit of 120s (Shiotsuki et al., 2010; Abdelsalam and Safar, 2015).

### Tissue preparation

One day after behavioral testing, the rats were anesthetized using intraperitoneal injection of pentobarbital (40 mg/kg). After intracardiac perfusion with phosphate buffered saline, the brains were rapidly dissected, placed on ice, and separated into two hemispheres without discrimination. The midbrain was dissected from one hemisphere and immediately preserved in RNA later for further analysis of gene and protein expressions. The other hemisphere was fixed by immersion in 10% paraformaldehyde for 24 h and processed by routine histopathological examinations using the paraffin method.

### Quantitative reverse transcriptase PCR

Total RNA was extracted from midbrain tissue according to a previous protocol (Chomczynski, 1993) using QIAzol reagent (Qiagen, Germany). The purity and concentration of RNA were detected by a NanoDrop 2000c Spectrophotometer (Thermo Scientific, United States). cDNA was synthesized using the COSMO cDNA synthesis kit (Cat. No. WF-10205002, Willowfort, United Kingdom) according to the protocol described by Wiame et al. (2000). The cDNA was subjected to an RT-qPCR assay using the Hera plus SYBR Green qPCR kit (cat. No. WF-10308001, Willowfort, United Kingdom) according to the manufacturer instructions. The following primers were used: Sirt1 Forward, 5′-GAGTTGTGTCATAGGTTAGGTGG-3′ and reverse, 5′-G TTAGAGGTCGCGC-CTACCAAGGCA-3′), *LC3* Forward, 5′-GTTAAGCCCCTACCAAGGCA-3′ and reverse, 5′-AGGGACTGTT TCCAGGGACT-3′ and glyceraldehyde 3-phosphate dehydrogenase (*gapdh*) Forward, 5′-AAGTTCAACGGCACAGTCAAG G-3′ and reverse, 5′-CATACTCAGCACCAG CATCACC-3′). The primer sets were synthesized by *Vivantis* Technologies (Malaysia). Real-time PCR was performed on a 20 μl reaction mixture using 7,500 real-time PCR Systems (Applied Biosystems) (95°C, 5 min), then 40 cycles (95°C, 10 s), and (60°C, 30 s). Sirt1 and LC3 expressions were normalized to the housekeeping *gapdh* gene and the relative expression levels of their mRNAs were determined using the (2^–ΔΔct^) method (Livak and Schmittgen, 2001).

### Western blot analysis

The extraction of total protein was performed with QIAzol reagent and its concentration was detected using the Bradford protein assay kit (Bio-Rad, United States). After mixing with loading buffer and heated for 8 min at 95°C, and then separated by electrophoresis of sodium dodecyl sulfate (SDS) polyacrylamide gel on a 12% gel followed by transfer to PVDF membrane. After blocking with 5% fat-free milk at room temperature for 2 h at room temperature, membranes were incubated with mouse monoclonal anti-MAP-LC3B (1: 1000, # Sc-271625, Santa Cruz, United States) and mouse monoclonal antiβ-actin (1:1000, # Sc-47778, Santa Cruz, United States), overnight at 4 C. Finally, the membranes were incubated with the corresponding goat anti-mouse IgG-HRP conjugated secondary antibody (1:4000, #sc-2031, Santa Cruz, United States) at room temperature for 2 h. The bound proteins were visualized using colorimetric immunodetection. Gels plugin of ImageJ software was employed to semi-quantify the protein bands on the membrane. The levels of the proteins studied were normalized to the β-actin.

### Histopathological examination

The formaldehyde-fixed midbrain samples were dehydrated in graded alcohol, cleared with xylol, and then fixed in wax. Paraffin sections of approximately 5 μm thick were obtained by a rotatory microtome.

(a) Staining with hematoxylin and eosin

Briefly, sections were deparaffinized in xylol, hydrated in descending grades of alcohol, stained with hematoxylin (H3136, Sigma-Aldrich) for 2 min, then washed in tap water for 10 min, stained in eosin (230251, Sigma-Aldrich) for 1/2 min, then washed in distilled water, dehydrated in ascending grades of alcohol, cleared by xylol and finally mounted in Canada balsam (Fischer et al., 2008).

(b) Immunohistochemistry

The sections were deparaffinized and rehydrated to distilled water then the slides were incubated with H_2_O_2_ (10%) for 15 min to block endogenous peroxidase. For antigen retrieval, the sections were immersed in a preheated citrate buffer solution (0.01 M, pH 6.0) in a water bath (95°C, 30 minutes). After that, sections were left to reach room temperature then incubated (1h) with BSA (1%) and then with primary antibodies; anti NF-κB p65 (1:100, # ab16502; Abcam, United Kingdom) (Yoshida et al., 1999), anti tyrosine hydroxylase (TH, 1:400, # ab137869, Abcam, United Kingdom) (Xu et al., 2020), anti-LC3 (1:200, # ab48394, Abcam, United Kingdom) and anti-P62 (1:200, # ab56416, Abcam, United Kingdom) (Erfan et al., 2021) overnight at 4°C. The nuclei were counterstained with hematoxylin. The image acquisition of stained sections was performed using the bright field mode of the Olympus^®^ CX41 light microscope connected to the Olympus^®^ SC100 digital camera. The experimental conditions were obscured during image acquisition and analysis to avoid bias. Equivalent fields in parallel midbrain sections from all groups were used for the analysis. The immunoreactivity was done using ImageJ software (Bethesda, MD, United States). The optical density of the TH, LC3 and P62 positive cells and the number of TH and NF-κB positive cells were evaluated per field (magnification: x400, area: 312.46 μm x 221.43 μm = 0.069 mm2). The number of TH-positive cells was determined using multipoint parameters in the interactive measurements menu in ImageJ. At least five non-overlapping fields from each slide were examined and the mean was calculated for each animal.

### Statistical data and analysis

Data were analyzed by SPSS-22 software (the statistical package for social science, version 22.0, IBM, Chicago, IL, United States). One-way analysis of variance (ANOVA) and Tukey *post hoc* tests were used to compare the experimental groups with assumptions of equal variance. Welch ANOVA and Games-Howell *post hoc* tests were used if the data violate the assumption of homogeneity of variance. Values are represented as mean ± standard error of the mean (SEM). Statistical significance was considered when *P* < 0.05.

## Results

### Calcitriol improved motor performance and exploratory behavior and anxiety in rotenone-induced Parkinson’s disease rat model

**FIGURE 1:**
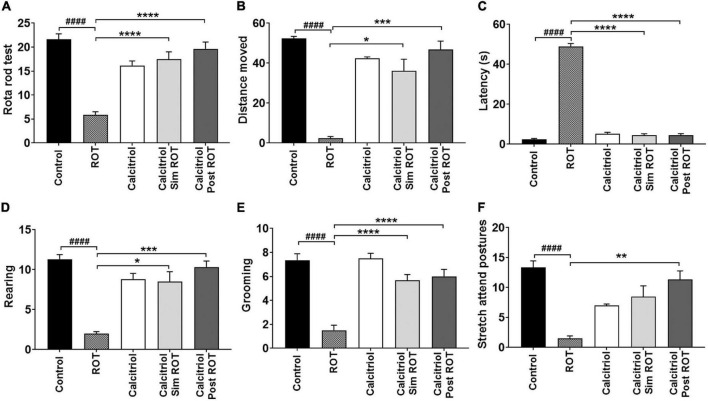
Behavioral analysis. **(A)** Rotarod test **(B–F)** Open field tests. Data are expressed as mean + SEM. One-Way ANOVA and *Post hoc* Tukey test [rotarod (*F* = 27.3, *P* < 0.0001), latency (*F* = 514, *P* < 0.0001), and grooming (*F* = 23.5, *P* < 0.0001)] and Welch ANOVA and *Post hoc* Games-Howell [rearing: F* (DFn, DFd) = 21.96 (4.000, 14.44), distance moved (cm): F* (DFn, DFd) = 35.85 (4.000, 9.927), and stretch attend posture: F* (DFn, DFd) = 15.19 (4.000, 14.25)]. ROT = rotenone, Calcitriol sim ROT group = simultaneous rotenone & calcitriol. Calcitriol post ROT group = calcitriol after 4 weeks of rotenone. *n* = 6 rats per group. *: *P* < 0.05, **: *P* < 0.01, ***: *P* < 0.001, ****: *P* < 0.0001 vs. ROT. ####: *P* < 0.0001 vs. control.

Rotarod test was used to evaluate motor performance and motor coordination. There was a statistically significant decrease in the ROT group compared to the control group (*P* < 0.0001). Either calcitriol sim ROT group or calcitriol post ROT treated groups showed a significant increase compared to the ROT group (*P* < 0.0001) (Figure 1A).

Motor performance was also evaluated by distance traveled and immobile latency in the open field. There was a statistically significant decrease in the distance moved in the ROT-induced PD group compared to control group (*P* < 0.0001). Calcitriol sim ROT and calcitriol post ROT groups showed significant increase in the distance traveled compared to the ROT group (*P* = 0.01; < 0. 001, respectively) (Figure 1B). Furthermore, the ROT group showed a significant increase in immobile latency compared to the control group (P < 0.0001) while calcitriol sim ROT and calcitriol post ROT groups showed a significant decrease in immobile latency as compared to the ROT group (*P* < 0.0001) (Figure 1C).

To evaluate the effects of calcitriol on exploratory behavior, the numbers of rears were counted. The number of rears showed a significant reduction in the ROT group compared to the control group (*P* < 0.0001). Both calcitriol sim ROT and calcitriol post ROT groups showed significant increases compared to the ROT group (*P* = 0.017; *P* < 0.001, respectively) (Figure 1D).

The effects of calcitriol on anxiety behavior were determined using grooming, stretching attending postures, and the number of central square entrants. The ROT group showed a significant decrease in grooming and stretch postures compared to the control group (*P* < 0.0001; *P* < 0.001). Calcitriol sim ROT and calcitriol post ROT groups showed significant increases in the number of grooming compared to the ROT group (*P* < 0.0001) (Figure 1E). In stretch attending posture, calcitriol post-ROT group showed a significant increase compared to the ROT group (*P* = 0.04), while calcitriol sim ROT group did not show significance compared to the ROT group (*P* = 0.05) (Figure 1F).

### Calcitriol improved the histopathological features and increased the expression of the TH in the midbrain of rotenone -induced Parkinson’s disease rat model

**FIGURE 2:**
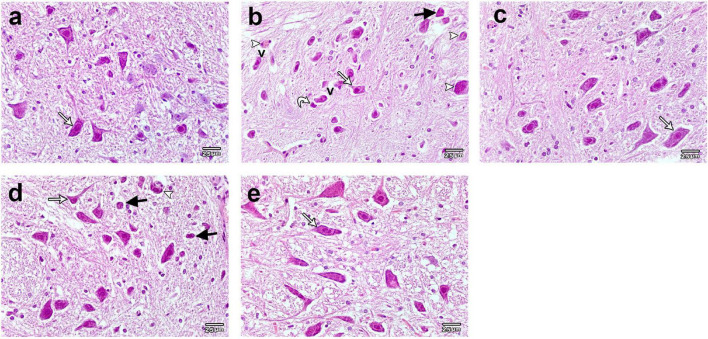
Representative photomicrographs of H&E stained sections of rats substantia nigra. **(a)** Control and **(c)** Calcitriol groups show dopaminergic neurons with vesicular nuclei and basophilic cytoplasm (white arrows). **(b)** ROT group shows neuronal loss, damage, and degeneration. Neurons appear smaller and shrunken (white arrow), many neurons illustrating irregular damaged cells, cytoplasmic shrinkage (curved arrow) and perineuronal vacuolations (V). Pyknotic darkly stained nuclei (black arrows), and cytoplasmic inclusions of Lewy bodies (arrowheads) are seen. **(d)** Calcitriol sim ROT group shows few degenerated neurons (black arrow), many normal appearing ones (white arrow), and cytoplasmic inclusions of Lewy bodies (arrowhead). **(e)** Calcitriol post ROT group shows improvement of the histopathological features with increased neuronal size (white arrow). Scale bar = 25 μm.

The midbrain of the control and calcitriol-treated groups exhibited a normal neuronal histological architecture with obvious vesicular nuclex‘i (Figures 2a,c). The midbrain of ROT-treated rats showed a marked decrease in neuronal cell number and size with obvious neuronal degeneration in the form of irregular damaged cells, cytoplasmic shrinkage, pyknotic nuclei, and chromatin condensation. The presence of Lewy body cytoplasmic inclusions, necrosis, and perineuronal vacuolation was also observed (Figure 2b). The midbrain of the calcitriol sim ROT group showed few degenerated neurons and many normal ones (Figure 2d). The midbrain of the calcitriol post-ROT group showed improvement in histopathological features and neuronal morphology (Figure 2e).

**FIGURE 3:**
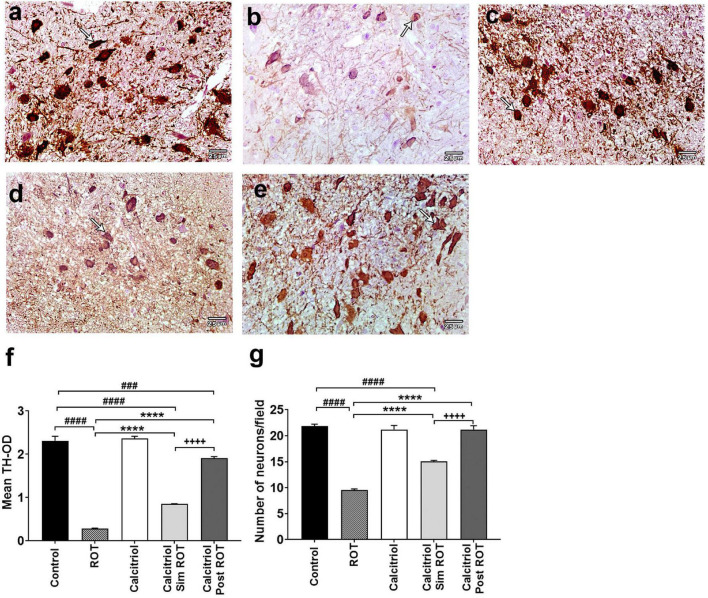
Expression of tyrosin hydoroxylase (TH) in rat substantia nigra. Representative photomicrographs of TH immunostained sections of rats’ substantia nigra from **(a)** control group and **(c)** calcitriol group showing strong TH immunoreactivity (arrows). **(b)** ROT group shows weak immunoreactivity and less number of DA neurons (arrow). **(d)** Calcitriol sim ROT group shows mild immunoreactivity (arrow). **(e)** Calcitriol post ROT group shows marked improvement in the TH immunoreactivity and increased DA neuronal number. Quantification of TH + cells showing the statistical differences in the **(f)** mean TH optical density (One-Way ANOVA: *F* = 281.6, *P* < 0.0001) and **(g)** number of neurons (One-Way ANOVA: *F* = 102.9, *P* < 0.0001) among the groups. OD: optical density. The above values are expressed as mean + SEM. n = 6 rats per group. ****: *P* < 0.0001 vs. ROT. ###: *P* < 0.001, ####: *P* < 0.0001 vs. control. + + + + : *P* < 0.0001 calcitriol sim ROT vs. calcitriol post ROT using One-Way ANOVA and *Post hoc* Tukey test. Scale bar = 25 μm.

To explore the effect of calcitriol on DA neurodegeneration in rats treated with ROT, the optical density (OD) was analyzed to evaluate healthy TH + DA neurons in the substantia nigra (SN) (Supplementary Figure 1). Treatment with ROT caused a significant decrease in TH-OD (0.289 ± 0.010) and the number of DA neurons (9.533 ± 0.531) compared to the control (OD: 2.313 ± 0.077, number: 21.867 ± 0.785) (*P* < 0.0001) (Figures 3a–c,f,g). The calcitriol sim ROT group provided a significant increase in DA neuron TH-OD (0.852 ± 0.011) and number (15.033 ± 0.566) compared to the ROT group (P < 0.0001). Furthermore, the calcitriol post ROT group resulted in a significant increase in DA neuron TH-OD (1.912 ± 0.036) and number (21.2 ± 1.089) compared to both ROT (*P* < 0.0001) and calcitriol sim ROT (*P* < 0.0001) groups. However, TH-OD in calcitriol sim ROT (*P* < 0.0001) and post ROT (*P* < 0.001) and number of TH + neurons in calcitriol post ROT (*P* < 0.0001) were still significantly lower than in the control group (Figures 3d–g).

### Calcitriol upregulated *Sirt1* expression in the midbrain of the rotenone-induced Parkinson’s disease rat model

**FIGURE 4:**
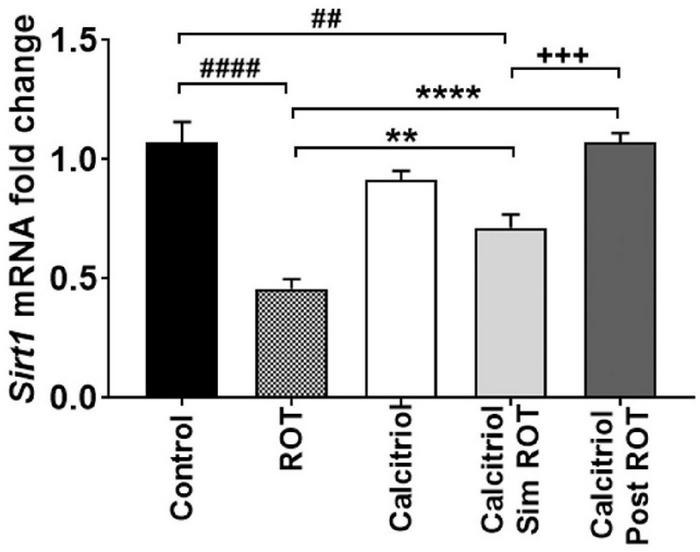
Quantification of *Sirt1* mRNA expression levels in midbrain of controls, ROT, Calcitriol, calcitriol sim ROT and calcitriol post ROT. mRNA fold changes was calculated relative to *gapdh* as housekeeping gene (One-Way ANOVA: *F* = 27.1, *P* < 0.0001). All data are expressed as mean + SEM. *n* = 3 rats in control group, *n* = 5-6 rats in other groups. **: *P* < 0.01, ****: *P* < 0.0001 vs. ROT. ##: *P* < 0.01 ####: *P* < 0.0001 vs. control. + + + : *P* < 0.001 calcitriol sim ROT vs. calcitriol post ROT using One-Way ANOVA and *Post hoc* Tukey test.

To evaluate the molecular mechanisms involved in the protective and restorative effect of calcitriol in the PD model, the expression level of the *Sirt1* gene was analyzed. Administration of calcitriol only to healthy rats did not alter their *Sirt1* expressions (0.93 ± 0.03, *P* > 0.05). However, our data showed that *Sirt1* expression is significantly reduced in the ROT group (0.46 ± 0.03) compared to control group (*P* < 0.0001). Administration of calcitriol simROT increased the reduced *Sirt1* expressions (0.71 ± 0.06, *P* < 0.01) though the expressions remained lower than the control group (*P* < 0.01). The administration of calcitriol post ROT also rescued the decreased expressions of *Sirt1* (1.07 ± 0.04, *P* < 0.0001) and with a statistically significant difference from the calcitriol sim ROT group (*P* < 0.001) (Figure 4).

### Calcitriol-induced autophagy and attenuated rotenone-induced autophagy dysfunction in the Parkinson’s disease rat model

**FIGURE 5:**
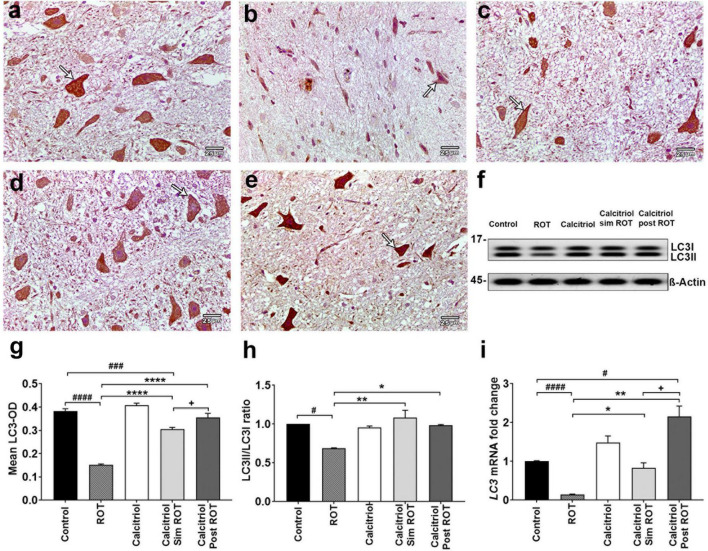
Expression of LC3 in rat substantia nigra. Representative photomicrographs of LC3 immunostained sections of rat substantia nigra of **(a)** control group and **(c)** calcitriol group showing strong LC3 immunoreactivity (arrows). **(b)** ROT group shows weak LC3 immunoreactivity and less number of DA neurons (arrow). **(d)** Calcitriol sim ROT group shows moderate LC3 immunoreactivity (arrow). **(e)** Calcitriol post ROT shows marked improvement in the LC3 immunoreactivity (arrow). **(f)** Western blot showing LC3I and LC3II expression in midbrain. **(g)** Quantification of mean LC3 optical densityin rat substantia nigra (*n* = 6 rats per group). **(h)** Quantification of LC3II/LC3I ratio in midbrain (*n* = 2 rats per group). **(i)** Quantification of *LC3* mRNA fold change in rat midbrain (*n* = 3 rats in control group, *n* = 5-6 rats in other groups). Data are expressed as mean + SEM. Scale bar = 25 μm. *n* = 6 rats per group. *: *P* < 0.05, **: *P* < 0.01, ****: *P* < 0.0001 vs. ROT. #: *P* < 0.05, ###: *P* < 0.001 ####: *P* < 0.0001 vs. control. + : P < 0.05 calcitriol sim ROT vs. calcitriol post ROT using One-Way ANOVA and *Post hoc* Tukey test (*LC3*-OD: *F* = 87.4, *P* < 0.0001, and western blot: *F* = 11.6, *P* = 0.009) and Welch ANOVA and *Post hoc* Games-Howell tests (*LC3* mRNA fold change F* (DFn, DFd) = 21.10 (4.000, 11.29).

We further confirm the effects of calcitriol on autophagy in ROT-induced PD model using the autophagosome marker *LC3* in immune-stained midbrain sections. LC3 illustrated positive cytoplasmic expression in the midbrain DA neuron of the control and rats treated with calcitriol (Figures 5a,c). The sections of the ROT group showed a significant decrease in LC3-OD (0.152 ± 0.003) compared to the control (0.383 ± 0.012) group (*P* < 0.0001) (Figures 5b,g). The immunoreaction of *LC3* was significantly increased in calcitriol sim ROT (0.305 ± 0.008) and calcitriol post ROT (0.355 ± 0.011) groups compared to the ROT group (*P* < 0.0001). LC3 expression in the calcitriol sim ROT group was significantly lower than in the calcitriol post-ROT (*P* = 0.03) groups (Figures 5d,e,g).

Western blot revealed that the ratio of LC3B-II/LC3B-I was decreased in the ROT group (0.7 ± 0.006, *P* = 0.02 versus the control group). Compared to the ROT group, the LC3II/LC3I ratio was elevated in the calcitriol sim ROT (1.08 ± 0.014, *P* < 0.001) and calcitriol post ROT (0.98 ± 0.02, *P* = 0.02) groups without statistically significant differences between these two groups (Figures 5f,h). Relative *LC3* mRNA expression levels in the midbrain revealed a significant decrease in *LC3* mRNA expression levels in the ROT group compared to the control group (*P* < 0.0001). In both the calcitriol sim ROT and calcitriol post-ROT groups, *LC3* expression levels were higher than in the ROT group (*P* < 0.05, *P* < 0.01; respectively). Additionally, calcitriol post was higher than the control group, as well as the calcitriol sim group (*P* < 0.05) (Figure 5i).

**FIGURE 6:**
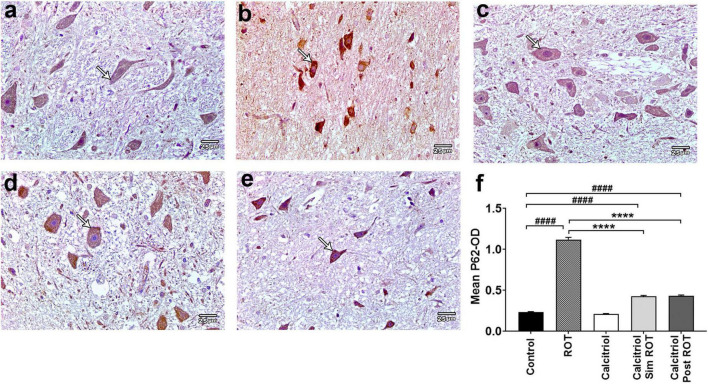
Expression of P62 in rat substantia nigra. Representative photomicrographs of P62 stained sections of rat substantia nigra from **(a)** control group and **(c)** calcitriol group showing very weak P62 immunoreactivity (arrows). **(b)** ROT group shows strong positive P62 immunoreactivity (arrow). **(d)** Calcitriol sim ROT group and **(e)** calcitriol post ROT group show mild P62 immunoreactivity (arrows). **(f)** Quantification of the mean P62 optical density (One-Way ANOVA: *F* = 703.5, *P* < 0.0001). The data is expressed as mean + SEM. *n* = 6 rats per group. ****: *P* < 0.0001 vs. ROT. ####: P < 0.0001 vs. control using One-Way ANOVA and *Post hoc* Tukey test. Scale bar = 25 μm.

Furthermore, the LC3 *binding* protein P62 exhibited minimal cytoplasmic immunohistochemical expression in both the control and calcitriol groups (Figures 6a,c). The ROT group revealed a strong expression of P62 that was significantly enhanced (1.121 ± 0.024) compared to the control (0.233 ± 0.006) (Figures 6b,f). The expression of P62 decreased markedly in both calcitriol sim ROT (0.427 ± 0.007) and calcitriol post-ROT (0.431 ± 0.008) groups compared to the ROT group (*P* < 0.0001) (Figures 6d–f). Taken together, these data suggest that calcitriol may be capable of inducing autophagy and restoring autophagy flux in the ROT-induced PD model.

### Calcitriol suppresses the inflammatory marker *NF-*κ*B* in the midbrain of the rotenone-induced Parkinson’s disease rat model

**FIGURE 7:**
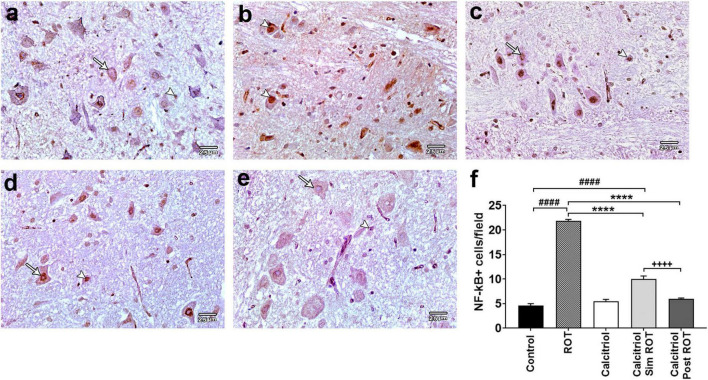
NF-κB expression in rat substantia nigra. Representative photomicrographs of NF-κB immunostained sections of rat substantia nigra from **(a)** control group and **(c)** calcitriol group showing occasionally seen positive nuclei (arrowheads) and many unstained ones (arrows). **(b)** ROT group shows higher number of positively immunostained nuclei (arrowheads). **(d)** Calcitriol sim ROT group shows mild immunoreactivity (arrowhead) together with negatively immunostained nuclei (arrow). **(e)** Calcitriol post ROT group shows marked reduction in the NF-κB immunoreactivity (arrow) and infrequently seen positive nuclei (arrowhead). **(f)** Quantification of NF-κB immunostained nuclei per field (One-Way ANOVA: *F* = 343.6, *P* < 0.0001). Data are expressed as mean + SEM. n = 6 rats per group. ****: *P* < 0.0001 vs. ROT. ####: *P* < 0.0001 vs. control. + + + + : P < 0.0001 calcitriol sim ROT vs. calcitriol post ROT using One-Way ANOVA and *Post hoc* Tukey test. Scale bar = 25 μm.

The expression of the NF-κB, a crucial inflammatory marker, was analyzed in the midbrain of all groups. Positive expression of NF-κB was observed in the cell nucleus. There were sporadically detected positive cells in the control (4.6 ± 0.27 cells/field) and calcitriol (5.5 ± 0.29 cells/field) groups, indicating very weak NF-κB immunoreactivity (Figures 7a,c). The ROT group showed an obvious increase in the number of NF-κB positive cells (21.87 ± 0.476 cells/field) compared to the control group (*P* < 0.0001). Compared to the ROT group, the number of NF-κB positive cells was strongly decreased in the calcitriol sim ROT (10 ± 0.457 cells/field) and post-ROT (6 ± 0.36 cells/field) (*P* < 0.0001) groups. Positive cells in the calcitriol sim ROT group were significantly higher than the calcitriol post ROT group (*P* < 0.0001). Additionally, NF-κB + cells in calcitriol sim ROT group remained significantly higher as compared to the control group (*P* < 0.0001) (Figures 7d–f).

## Discussion

Current dopamine replacement therapies for PD provide relief from some disease symptoms; however, their effectiveness wears off over time and their long-term use results in serious side-effects for PD patients (Hegarty et al., 2016). Therefore, the establishment of novel therapeutic approaches is urgently required. Although calcitriol is initially known for its importance in calcium homeostasis and metabolism, it is recently associated with multiple pathological disorders, including PD (Fullard and Duda, 2020). Current evidence suggests a transposed correlation between calcitriol levels and the risk of developing PD (Lv et al., 2020). In addition, the vitamin D receptor and 1α-hydroxylase, the enzyme responsible for converting vitamin D to its active form (calcitriol), are strongly expressed in SN (Eyles et al., 2005). These findings support the assumption that impaired vitamin D levels may result in malfunction or increased cell death within the SN (Fullard and Duda, 2020). The pathophysiology of PD is affected by calcitriol through genetic and non-genetic routes (Ebert et al., 2006). Calcitriol can enhance or decrease the expression of several genes, thus affecting intracellular signaling pathways (Lv et al., 2020). Our study reported that calcitriol significantly decreased ROT-induced motor impairment, neuroinflammation, and autophagy dysfunction in the midbrain of the rat model of PD presumably due to calcitriol-upregulated *Sirt1* expression that could in turn with subsequent stimulation of autophagy and inhibition of NF-κB.

In the current study, ROT treated rats showed a significant dysfunction in locomotion and coordination. Consistent with our findings, ROT-treated animals showed motor abnormalities in the open field test (Zaitone et al., 2012). Our results of open field tests suggested calcitriol supplementation improves motor performance in rats, in agreement with Sakai et al. (2015) who showed that 1,25-dihydroxyvitamin D enhanced locomotor performance in mice. Calcitriol supplementation improved muscle strength and physical activity (Girgis et al., 2014; Wyon et al., 2016). Our results revealed that calcitriol affected balance and coordination as assessed by the rotarod test. We demonstrated an improvement in rotarod and immobile latency. These results were in agreement with Seldeen et al. (2020) who confirmed that mice showed better rotarod performance after three months of calcitriol treatment.

Parkinsonian rats injected with ROT displayed significant dopaminergic neurodegeneration in SN represented by decreased TH-immunopositive neurons. Consistent with our findings, Betarbet et al. (2002) reported that ROT-treated animals caused nigrostriatal neuronal loss. Furthermore, Zhang et al. (2019) showed that the number of TH-positive neurons in the normal brain hemisphere was twice the number of TH-positive neurons in the damaged brain hemisphere in a 6-hydroxy-dopamine Parkinsonian mouse model. TH is a rate-limiting enzyme for dopamine synthesis and, interestingly, may be directly regulated by calcitriol, which is also consistent with our observation by immune-histochemical staining. In line with this, Lima et al. (2018) found that calcitriol increased immunostaining of TH in hemi-parkinsonian. Cui et al. (2015) showed that application of calcitriol, before or after local 6-hydroxydopamine injury, relatively restores TH protein expression levels and TH-immunoreactive fibers in the striatum and SN. Although it is a matter of debate whether dopaminergic neurogenesis occurs in the adult midbrain, some reports showed that dopaminergic neurons are capable of regeneration in the salamander model of PD, probably by activating stem cells along the ependyma of the third ventricle and/or reprogramming of differentiated cells. Consequently, this restoration of dopaminergic cells could contribute to the behavioral recovery (Parish et al., 2007). However, further future studies are required to address this possibility in mammals.

Sirt1 is strongly expressed in both neurons and glial cells in the human brain (Koronowski and Perez-Pinzon, 2015), as well as in the adult mouse brain (Zakhary et al., 2010). It is also expressed in the nucleus and cytoplasm of dopaminergic neurons and microglial cells in SN of rats (Diaz-Ruiz et al., 2015). Its expression is reduced due to aging and some neuropathological changes (Quintas et al., 2012). Although there are very limited studies correlating PD with *Sirt1* expression, α-synuclein protein aggregation reduces Sirt1 expression (Manjula et al., 2020), which may explain why patients with PD are particularly susceptible to neurotoxin-induced neuronal damage (Li et al., 2020). Our study demonstrates that ROT down-regulates *Sirt1* expression, which is in agreement with Tao et al. (2020) who showed that *Sirt1* levels were decreased in ROT-treated animals and *in vitro* models of PD.

However, previous reports have already documented that *in vitro* calcitriol upregulated *Sirt1* and reversed *Sirt1* down-regulation in human umbilical vein endothelial cells exposed to H_2_O_2_ oxidative stress (Polidoro et al., 2013). Chang and Kim (2019) revealed that vitamin D insufficiency significantly reduced *Sirt1* mRNA expression in induced obese rats, while vitamin D supplementation restored *Sirt1* transcription levels. Consistently, our results reveal that although the application of calcitriol in healthy rats had no effect on *Sirt1*, calcitriol supplementation in combination with ROT for 4 weeks rescued the expression of *Sirt1, although it was* lower than the control group. This may be explained by the long-lasting inhibitory effect of ROT on *Sirt1* expressions. In addition, calcitriol administration for eight days after PD induction also up-regulates *Sirt1*. However, our data limit to gene expression level, while Sirt1 protein level as well as *Sirt1*-related activation markers of mitochondrial biogenesis, remain to be examined in future studies.

Dysregulation of autophagy has been involved in a wide range of environmental chemical-induced neurotoxicity (Zhou et al., 2017). Furthermore, strong evidence indicates that dysfunctional autophagy leads to the accumulation of abnormal proteins, including α-synuclein (Ghavami et al., 2014), or accumulation of damaged cell organelles in PD models (Xiong et al., 2013). Impairment of autophagy has been demonstrated in patients with Lewy body disease and in a model of α-synucleinopathy. It has been reported that α-syn can interfere with the early stages of autophagosome formation. Thus, accumulation of α-syn is cytotoxic for neurons and can also cause a disruption of the autophagy process, leading to even faster protein accumulation (Rakowski et al., 2022). The present study employed ROT as an environmental chemical neurotoxin and is capable of inducing PD in rats (Betarbet et al., 2000).

LC3 is widely recognized as an autophagic indicator to assess autophagy activity of autophagy as it is present in the membrane of the autophagosome (Kabeya et al., 2000). Furthermore, P62 is an autophagy receptor that interacts with ubiquitinated cargo and LC3 (Rogov et al., 2014) and is considered a hallmark of autophagic flux, during which P62 is constantly degraded (Pankiv et al., 2007). Thus, a high LC3/low P62 suggests activated and intact autophagy, while a low *LC3/p62* may show low basal autophagy (Liu et al., 2014).

Here, we demonstrate down-regulation of LC3 gene and protein expressions, along with an increase in *P62* level in the midbrain of ROT-treated rats signifying a suppressive effect of ROT on autophagy pathways. Our results were in agreement with the previous finding showing that ROT administration resulted in decreased LC3 and Beclin-1 expression, and increased P62 protein level (Zeng et al., 2019).

Recent reports have shown that vitamin D supplementation elevates basal levels of autophagy and reduces the parameters of oxidative stress (Sepidarkish et al., 2019). Serum levels of vitamin D in PD patients have been found to be approximately two times lower than in healthy subjects, suggesting that there may be a correlation between vitamin D level and autophagy in PD (Ogura et al., 2021). Interestingly, vitamin D modulates autophagy at several levels through different mechanisms, including the regulation of intracellular calcium levels and its downstream pathways (Abdel-Mohsen et al., 2018). Tavera-Mendoza et al. (2017) reported that the vitamin D receptor (VDR) acts as a master transcriptional regulator of autophagy. They observed that vitamin D supplementation induces autophagy in the normal murine mammary gland through up-regulation of the LC3 protein level accompanied by increased autolysosome volume. Our results added evidence that calcitriol administration induced both an increase in LC3 expression and a decrease in *P62* expression in ROT-treated rats. Surprisingly, the expression of the LC3 gene was remarkably upregulated when vitamin D was administered post-ROT than the control rats. This may indicate persistent compensatory up-regulation of the LC3 gene. This increase was not reflected at the functional protein level, probably due to post-translational modification (Lindner and Demarez, 2009). However, this hypothesis needs to be tested experimentally.

Thus, to further study the effect of calcitriol on autophagic activity in PD, we measured the conversion of the soluble cytosolic form of LC3 (LC3I) to the lipidated and autophagosome-associated form (LC3II) (Tanida et al., 2008) by Western blot. We found that the LC3II/LC3I ratio was significantly increased upon administration of calcitriol to rats treated with ROT, indicating intact autophagy and increased autophagosome abundance. Our findings are consistent with previous study of Jang et al. (2014) that demonstrated that calcitriol protects against ROT-induced neurotoxicity in SH-SY5Y cells by improving autophagy signaling pathways such as those involving LC3 and beclin-1.

Importantly, calcitriol-induced *Sirt1* up-regulation observed here may support activated autophagy. This is in line with previous studies that demonstrate that *Sirt1* plays a central role in the promotion of autophagy through the deacetylation of autophagy-related proteins, including LC3 and by stimulating the conversion of LC3-I to LC3-II through proteolytic cleavage and lipidation (Lee et al., 2008). Furthermore, Sirt1 activation causes translocation of LC3 from the nucleus into the cytoplasm, which is conveyed by increased expression of LC3-II and of α-synuclein and P62 degradation in dopaminergic neurons (Guo et al., 2016).

Taken together, these findings suggest that the calcitriol effect is related to the induction of autophagy and the restoration of autophagy flux and, therefore, the inhibition of autophagy dysfunction in the midbrain of the PD rat model.

Although neuroinflammation might exert a protective effect on the CNS (Wee Yong, 2010), several studies advocate the hypothesis that neuroinflammation plays an essential role in the pathogenesis of PD (Anusha et al., 2017). Dysfunctional mitochondria that accumulate due to impaired autophagy may play a role in initiating this neuroinflammation (Berridge, 2017). NF-κB is a key player in inflammation and regulates several other inflammatory markers (Hajiluian et al., 2018). Activation of NF-κB leads to its translocation to the nucleus and stimulates the transcription of genes that encode multiple pro-inflammatory factors (Zhang et al., 2010). Up-regulation of NF-κB is generally associated with neurodegeneration (Anusha et al., 2017) and promotes apoptosis in patients with PD (Holmes et al., 2016). In the present study, we found that the NF-κB was activated in the midbrain of rats treated with ROT, which is in agreement with previous observation (Thakur and Nehru, 2015).

Interestingly, vitamin D may modulate inflammation through down-regulation of pro-inflammatory factors and, presumably, could improve cognition (Vanoirbeek et al., 2011). Furthermore, the expression of vitamin D receptors, 1α and 24α-hydroxylase in various cells of the CNS advocate its pivotal role in central inflammation (Erbaş et al., 2014). Calcitriol ameliorates pro-inflammatory effects by modulating cytokine production or release, which might lead to restoration of cellular homeostasis to prevent subsequent organ damage (Tsai et al., 2016). Consistent with our findings, calcitriol administration to ROT-treated rats significantly diminished NF-κB expression, with a stronger effect when calcitriol was administered after stopping ROT treatment. This can be explained by rotenone stimulation of intracellular reactive oxygen species generation, which then promotes the NF-κB activation and translocation to the nucleus (Yuan et al., 2013). This inhibition of NF-κB shows that calcitriol has anti-inflammatory activity, which may slow the progression of PD. Previous investigations, in line with our findings, revealed that vitamin D may down-regulate NF-κB expression in inflammatory diseases, for example, multiple sclerosis (Cantorna et al., 1996), type 1 diabetes (Mathieu et al., 1995), and inflammatory bowel disease (Lim et al., 2005).

In addition, calcitriol is capable of modulating NF-êB and its related inflammatory pathways in dendritic cells (D’Ambrosio et al., 1998), lymphocytes (Yu et al., 1995), fibroblasts (Harant et al., 1998), and keratinocytes (Riis et al., 2004). Furthermore, a vitamin D analog is shown to strongly suppress the production of pro-inflammatory chemokine in pancreatic islet cells, which is accompanied by an enhancement of IκBα transcription and a stop of NF-κB P65 nuclear translocation (Giarratana et al., 2004). It is noteworthy that pro-inflammatory cytokines, which are closely linked to PD including IL-1ß, IL-6, and TNF, remain to be investigated to better elucidate the potential role of calcitriol in modulation of neuroinflammation in PD models (Nagatsu and Sawada, 2005).

Importantly, calcitriol might exert its anti-inflammatory effect through the Sirt1/NF-κB signaling pathway (Li et al., 2020). Vitamin D supplementation upregulated *Sirt1*expression and decreased NF-κB phosphorylation (Safarpour et al., 2020). In addition, NF-κB has emerged as a negative regulator of autophagy (Djavaheri-Mergny et al., 2006). Taken together, these results suggest that calcitriol administration leads to decreased neuroinflammation in the midbrain of PD rat model. Interestingly, we showed improvement in some aspects including higher TH neurons, increased Sirt1 and LC3 expression, and decreased NF-κB cells when calcitriol was administered after ROT administration. This may indicate that the calcitriol may have a stronger therapeutic effect than its role in neuroprotection, and the beneficial effect of calcitriol could be maintained even after the onset of the pathology.

In conclusion, our data point to a prophylactic and restorative effect of calcitriol on motor and exploratory behaviors, as well as neuronal architecture, autophagy, and neuroinflammation in the ROT-induced rat model of PD, presumably through *Sirt1*-dependent mechanisms. Given this association between calcitriol and Sirt1 expression, calcitriol could be a target that protects against or improves the manifestation of PD at both the functional and structural levels. However, it is of great importance to elucidate whether calcitriol treatment decreases the α-Syn protein aggregation in PD, affects other Sirt1-related pathways, or modulates neuroinflammatory endpoints, including cytokines, in future studies.

## Data availability statement

The raw data supporting the conclusions of this article will be made available by the authors, without undue reservation.

## Ethics statement

The animal study was reviewed and approved by Ethical committee, Faculty of Medicine, Mansoura University, Egypt.

## Author contributions

AM, EF, SH, ZA, and ME-k performed the material preparation, data collection, and analysis. AM contributed to the project administration. EE and MA acquired the funding and contributed to the study conception and design. AM, EF, AA, and ME-k performed the visualization and wrote the first draft of the manuscript. AM, EF, SH, ZA, EE, MA, AA, and ME-k commented on previous versions of the manuscript and read and approved the final manuscript.
