# Supplementary material for: Neuroprotective and therapeutic effects of calcitriol in rotenone-induced Parkinson’s disease rat model
Source: Front Cell Neurosci. 2022 Sep 16;16:967813. doi: 10.3389/fncel.2022.967813 (PMC9522903; doi:10.3389/fncel.2022.967813)
Supplement: Supplementary file 1 [file Data_Sheet_1.docx]

Supplementary Material

## Supplementary Figures

**
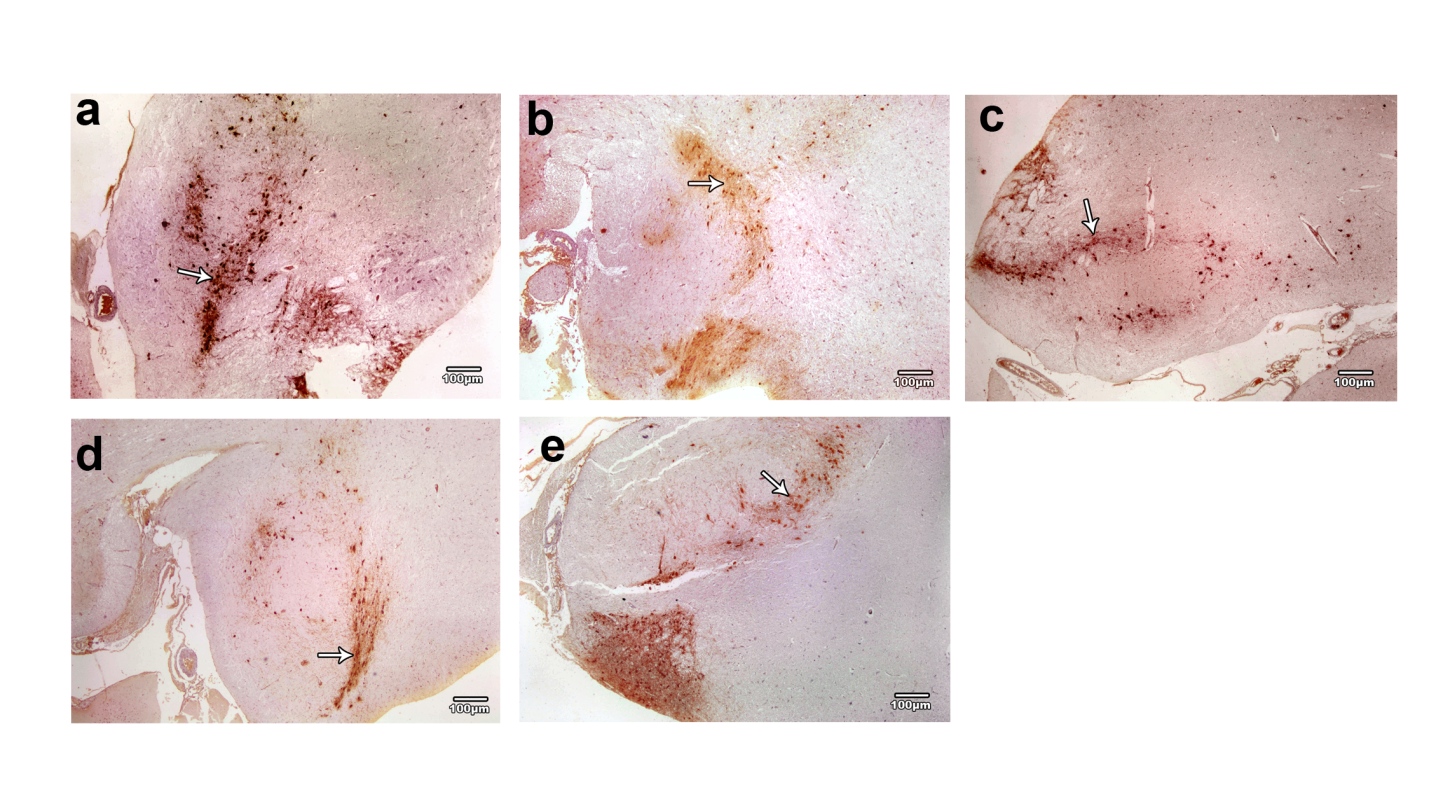
**

**Supplementary Fig. S1:** Representative overview photomicrographs of a) Control, b) ROT c) Calcitriol, d) Calcitriol sim ROT, and e) Calcitriol post ROT rat midbrain showing TH+ neurons in substantia nigra (arrows). Scale bar = 100µm.
